# The Poor-Law Medical Service

**Published:** 1915-09-04

**Authors:** 


### THE POOR-LAW MEDICAL SERVICE.

### Its Hospital Branch.

The valuable opportunities for increasing his practical experience presented to the newly-qualified man by the medical service of the Poor Law are not sufficiently recognised. This is largely due to the prejudice which still exists against everything associated with the Poor Law. The medical profession, as well as the public generally, is ignorant of the great advances made in the medical branch of the Poor Law during the last thirty years. Great as these advances have been, it must not be inferred, however, that the position to-day is perfectly satisfactory. The infirmaries are all understaffed both as regards medical men and nurses, and there is an almost complete absence of specialist appointments. For some little time prior to the war, and more especially since the war, the scale of remuneration attached to the resident medical posts has gradually risen owing to the great difficulty in obtaining candidates. The scale varies considerably, but as a rule it may be said that in an infirmary with three assistant medical officers the junior will be paid from £150 to £200, the second from £200 to £250, and the senior from £250 to £300 per annum, with the usual residential allowances. In most cases these salaries have been sanctioned by the Local Government Board for the period of the war only, but there is little doubt that the scale of remuneration will not again sink to the low levels ruling five years ago. The salaries paid to the medical superintendents also vary very much. Taking into consideration emoluments and extra fees, it may be said that these posts vary in value from £600 to £900 per annum, the higher figure being attained in only a few instances. The post of medical superintendent is usually, but by no means always, filled by promotion from the ranks of the junior medical officers, but vacancies occur at irregular and often at long intervals, so that the chance of obtaining one is not great.

### Its Many Attractions.

The indoor Poor-Law Medical Service is therefore not one which offers many attractions to a man seeking a permanent opening. It presents, however, many advantages to the newly-qualified man who wishes to devote profitably two or three years to extending his experience and applying practically the knowledge he has acquired in his student days before settling down to private practice. Such a man will find the patients in a Poor-Law infirmary present him with a field of work which is inadequately represented at most voluntary hospitals. He will see bedridden patients in the advanced stages of rheumatoid arthritis, gout, and chronic nervous diseases; the terminal stages of inoperable malignant disease and of phthisis; the various pathological conditions associated with senile decay; childish infectious disorders, such as whooping cough, measles, and German measles; and the marasmic disorders of infancy. Cases such as these, which the medical student usually sees only in the out-patient department or for a few days in the wards, he will now be able to study over long periods and will learn the difficulties of treating them, the pitfalls in the way of accurate diagnosis, and the effects of treatment in curing or alleviating their symptoms. The maternity wards, the lock wards, and the insane wards will enable him to study other phases of medical practice which will be of inestimable value to him in his career as a medical practitioner. The work in the insane wards is of special value to the newly-qualified man, because here he sees cases of incipient and doubtful insanity, and learns practically the details the law requires to be observed in the certification of the insane.

### Its Educational Value.

Another educational advantage of these residential appointments is the fact that the holder of them finds that he is expected to rely upon himself. He will act, of course, under the general supervision of the medical superintendent, and will be encouraged to consult with him and his senior colleagues in matters of difficulty, but the treatment of the cases has necessarily, as a rule, to be left in his hands. He will be required to carry out special methods of investigation himself. He will have many opportunities of using such instruments as the laryngoscope, the ophthalmoscope, the sphygmomanometer, etc.; he will be expected to familiarise himself with the use of the *x*-ray apparatus, to employ electricity for therapeutic and diagnostic purposes, and to apply the ordinary chemical and microscopical methods for the examination of blood, urine, etc. He will gain experience in giving anæsthetics, the minor surgical work will be left in his hands, and he will be required to assist at the major operations. The work is therefore just of that character which will be of most value to him later in life as a general practitioner. The clinical material is so rich and varied that he will have many opportunities for research work.

A Word in Season.

It is necessary, however, that care should be taken in applying for a post in a Poor-Law infirmary. These infirmaries vary very much as regards both equipment and the character of the work done in them. The best work is only to be obtained in the separate infirmary under the administration of a medical superintendent. As a general rule, to which there are a few exceptions, infirmaries which are under the administration of the workhouse master should be avoided. The intending applicant should make inquiries as to the equipment, the number of beds, the number of admissions annually, and the number of operations performed. The best infirmaries possess a pathological laboratory, *x*-ray apparatus, an electrical department, and an operating theatre. In them two or three hundred or more operations will be performed each year, the number of admissions will be about five or six times the average number of occupied beds, and the medical officer will not be required to do the dispensing or any clerical work beyond clinical note-taking.

War Hospital Changes.

During the last six months many Poor-Law infirmaries have been taken over by the War Office and used as hospitals for sick and wounded soldiers. The ordinary inmates thus displaced have been accommodated in the infirmaries of neighbouring unions. The Guardians' staff has usually been taken over by the War Office. In London the medical superintendent has been given the rank of major, and the whole-time medical staff the rank of lieutenant. Outside London the rule has been to make the medical superintendent a lieutenant-colonel. These officers have been given either the salary and extra fees they have received from the Guardians in the year previous to the change or a salary on the War Office scale, whichever is the higher. The medical staff has been considerably increased by the appointment of commissioned officers of the R.A.M.C. and of civilian practitioners and specialists. In London the regular staff usually includes a radiographer, an ophthalmic surgeon, one or more operating surgeons, a neurologist, and a dental surgeon. In addition the hospitals have been supplied with a list of specialists in all the chief departments, who have volunteered to attend whenever required. The nursing staff has also been largely increased by the addition of trained nurses, probationers belonging to Voluntary Aid Detachments, and orderlies. The nurses are paid at War Office rates, which are much higher than the Poor-Law scale. Sisters are paid at the rate of £50, rising to £65, and staff nurses £40 rising to £45, with £8 per annum for dress allowance. The type of case in these infirmaries has, of course, completely altered. The cases are mainly surgical, and the number of operations has considerably increased. There are very few bed-ridden cases, and there is a considerable increase in the number of convalescent patients, and of those who are able to be up all day. The diseases treated form a much less varied group than in the old days. The operative work, too, is less varied, consisting mainly of operations for the removal of foreign bodies or for the treatment of septic wounds, of radical cures for hernia, varicocele, hydrocele, and varicose veins, of nerve suture, bone plating, and other operations for the repair of injured tissues. Children and women are no longer treated, and the result is that the training of probationers is far less satisfactory than formerly. It is, in fact, a question whether these infirmaries may not have to give up temporarily the training of probationers, and replace them by Red Cross probationers. One result of the war has been to show that infirmary accommodation is greater than is necessary for the treatment of the sick under the Poor Law. It is possible that, as a result of the experience that has been gained, many Poor-Law institutions throughout the country will be closed when the war is over, and the remaining institutions more fully utilised. An impetus will undoubtedly be given to the movement for the amalgamation of Poor-Law areas so as to enable this to be done with greater ease. The result would be an increase in efficiency and economy.

